# Analysis and assessment of biomedical scientists’ needs for clinical laboratory: activity-based management as an evaluation methodology

**DOI:** 10.3389/fbioe.2025.1569800

**Published:** 2025-05-13

**Authors:** Claudia Bizzoni, Gavino Napolitano, Simonetta Cesa, Luca Sacella, Caterina Bianciardi, Cosimo Ottomano, Rita Mancini, Giorgio Da Rin

**Affiliations:** ^1^ Department of Laboratory Medicine, ASST Papa Giovanni XXIII, Bergamo, Italy; ^2^ Strategic Steering Commitee, Centro Studi SAPIS Foundation, Italian National Federation of Orders of Radiographers and Technical, Rehabilitation, and Prevention Health Professions Research Centre, Rome, Italy; ^3^ ASST Papa Giovanni XXIII, Bergamo, Italy; ^4^ SYNLAB Italia Srl, Brescia, Italy; ^5^ IRCCS Policlinico Sant'Orsola, Bologna, Italy; ^6^ Chief Laboratory Medical Officer, SYNLAB Italia Srl, Monza, Italy; ^7^ Clinical Laboratory Director LUM - Laboratory Medicine, Maggiore Hospital, Bologna, Italy; ^8^ Department of Biomedical Sciences, Humanitas University, Milan, Italy

**Keywords:** activity based management, staffing needs, clinical laboratory, biomedical scientist, management of healthcare organizations

## Abstract

**Introduction:**

Healthcare systems have to protect citizens’ health by developing models combining concepts of efficiency, effectiveness and quality of care. The post-Covid-19 pandemic context has highlighted the relevance of efficiently managing and allocating human resources. In this scenario, the analysis and calculation of personnel needs take on strategic importance. The project aims to suggest a methodology to define the needs of Biomedical Scientists. The goal is to create a standard model adaptable to different contexts.

**Methods:**

This project, developed in cooperation with the Italian Society of Clinical Biochemistry and Clinical Molecular Biology, has created a new format following the “Activity Based Management” approach. It is characterized by continuous improvements, based on analysis of processes, broken down into sub-processes and activities. After the phase of format development, a phase of application to different contexts, such as biochemistry and the hematology sectors, followed.

**Results:**

The suggested methodology allows to estimate the number of Full Time Equivalents necessary for the management of the laboratory processes. Furthermore, an objective and analytical data is obtained, because it is based on timely numerical surveys that included productivity and execution times of the different activities.

**Discussion:**

Using the format had a relevant impact on the analysis of the processes, their efficiency, and their possible improvement. This method allowed to evaluate and improve the analytical and “extra-production” activities, often underestimated but having a decisive role in the process. The proposed format can be considered a valid tool for laboratory managers to analyze and evaluating the needs of Biomedical Scientists in the laboratory. Activity Based Management allowed us to obtain precise and objective data and, at the same time, to focus on the main objective of any clinical laboratory: to create value for the patient by supporting diagnosis and treatment of paths through safe and reliable laboratory tests, which depends on a correct allocation of human resources.

## 1 Introduction

The management of healthcare organizations, due to the specific political and economic context in which they are currently operating (Ministry of Economy and Finance, 2000), is required to ensure a balance between the need to make the best use of economic resources and, at the same time, the need to respond to a growing demand for healthcare services and to pursue improvements in quality of care and assistance. This has led to the development of models combining concepts of efficiency, effectiveness, and quality. Although there is a wide range of factors that can influence organizational performance, such as organizational structure, technology employed and strategy conducted, their impact is less than that generated by the human resource management system adopted (Marchington and Grugulis, 2000). Accordingly, the workload analysis takes on strategic importance. In addition, the Covid-19 emergency has strongly highlighted the issue of staffing requirements, so a proper management of available human resources and their optimal allocation are necessary to safeguard quality of care and to achieve greater efficiency (Napolitano et al., 2021). Several studies show a direct correlation between staffing and care outcomes (Sasso et al., 2017; Aiken et al., 2014), proving that an adequate staffing level is needed to ensure the quality of care and patient safety. For nursing profession, there are several studies and methods that have been tested to appropriately measure workloads (Griffiths et al., 2020). On the other hand, for the profession of Biomedical Scientist, among the current models for defining staffing needs in healthcare, there is no model to be considered as a gold standard or specifically dedicated to the profession. There are models developed by the World Health Organization (WHO) in 1998 and taken up in 2010 and 2016 (Workload indicators of staffing need WISN) (Stankovic and Santric Milicevic, 2022; Tripković et al., 2022; Doosty et al., 2019), by the Organization for Economic Cooperation and Development (OECD) in 2013 (Health workforce requirements for universal health coverage and the sustainable development goals human resources for health observer) (Scheffler et al. 2016), and by the European Union in 2014 (User guidelines on qualitative methods in health workforce planning and forecasting) (Kroezen et al., 2018). However, none of these reflect the specificity and complexity of the profession of the Biomedical Scientist. Laboratory Medicine today plays an extremely important role in disease prevention, diagnosis, monitoring of disease progression, and evaluation of outcomes (Körber and Plebani, 2013) (Olver et al., 2022). Consequently, the performance and quality of Laboratory Medicine Services are crucial to ensure safe, appropriate, efficient, and effective care for patients (Plebani et al., 2019). Recent years, especially with the advent of the COVID-19 pandemic, have increasingly brought to light the dominant role of Laboratory Medicine in clinical reasoning (Lundberg, 1981; Plebani et al., 2011; Lundberg, 2014; Favresse et al., 2022). This growing importance has led to an ever-increasing demand for services, new test implementation, quality improvement and reduction of Turn Around Times (Plebani, 2018a) (Plebani, 2018b).

However, increased productivity and introduction of new diagnostics, aimed at meeting clinical - care needs, cannot ignore the effects these activities have on the quality of current services provided if not accompanied by adequate human resources (Plebani, 2009). The huge demand for human resources in healthcare, in this specific case of Biomedical Scientist, collides with the economic pressure on cost containment that clinical laboratories are facing in recent years. In this context, there is a lack of an accurate methodology for estimating the need for Biomedical Scientist personnel in laboratories. The literature on the subject is poor, with only few benchmarking studies (Novis et al., 2022; Jones et al., 2012; Valenstein et al., 2005). Even analyzing the accreditation requirements, there is no guidance for accurately calculating staffing requirements, often defining only a minimum value for Biomedical Scientists (Lombardia, 2005; Decreto Ministeriale 24.01, 2023). The urgency of finding a shared methodology is certainly a current topic of interest to clinical laboratory managers, who have always had difficulty in formulating staffing needs because of the complex nature of laboratory activities. Using uniform staffing standards that do not take into account the type of work performed within the laboratory, can create problems in providing for the required services. Different laboratories vary widely in many respects, including complexity of the context they are located; number of services performed and related complexity of execution; organizational model adopted; opening hours for service guarantee (h24/h12/h8) and need for reduced Turn Around Time (routine or urgent regime) (Dawande et al., 2022). Not least, laboratories may differ, even considerably, in the degree of technological advancement and automation (Sarkozi et al., 2003). However, while the effects of Total Laboratory Automation (TLA) on productivity, cost reduction, and service quality improvement have been studied (Al Naam et al., 2022), little data are available regarding its impact on the personnel needed in the laboratory. Despite the expansion of TLA, the Biomedical Scientist continue to play a vital role in the clinical laboratory.

This project aims to suggest a methodology for defining the need of Biomedical Scientists. As part of this project, we want to build a standard model that will allow us to meet the needs of laboratory directors and technical coordinators, providing them with a timely management tool to estimate staffing based on objective data for informed decision making. Once the format was elaborated, it was applied to the biochemistry/immunometry and hematology sectors of the Corelab area of the Clinical Chemical Analysis Laboratory of the ASST Papa Giovanni XXIII in Bergamo, a modern laboratory with very high automation level, that handles a considerable amount of samples by offering an active H24 service (Napolitano and Parimbelli, 2022).

## 2 Materials and methods

The methodology proposed in this project involves the compilation of a format, developed in cooperation with the Italian Society of Clinical Biochemistry and Clinical Molecular Biology (SIBioC), one of the most relevant Italian scientific societies of Laboratory Medicine. The working group was made up of doctors, biologists, Biomedical Scientists and engineers. This highlighted how multi-professionalism and multidisciplinarity in laboratory setting are necessary today (Napolitano and Vitullo, 2023). Laboratory Medicine is no longer a mere service ready to give answers by simply performing laboratory tests, but it is an extremely complex reality, populated by different professionals who, with different and complementary skills, play a key role in the decision-making process of the clinician (Sandhaus, 2012).

The developed format follows the “Activity Based Management” (ABM) approach. It is based on the identification and evaluation of processes, broken down into sub-processes and activities. ABM is directed to improve the effectiveness and efficiency of management processes and to understand how complexity can affect the impact of resources on business objective (Ostinelli, 1995).

Through the ABM system it is possible to activate continuous improvement logics based on the constant monitoring of processes and the identification of actions for their improvement, to which it adds stringent criteria of measurability (Kaplan and Anderson, 2004; Keel et al., 2017). The assumption from which it started is a proper balance between production efficiency and qualitative effectiveness in the laboratory: there is a need to optimize and reduce costs while ensuring the quality of the service provided. As such, this model seeks to maintain this important balance.

The format was developed for two areas of the laboratory: biochemistry/immunochemistry and hematology. The file, for each of the two analytical lines, is structured into three spreadsheets to be completed.

1. Mesophase time entry
2. Official coding
3. Extra Production Activity

The “Mesophase Time Entry” sheet shows the macroprocesses, broken down into subprocesses, in which all activities, from the pre-analytical phase to the post-analytical phase, have been listed and grouped. It is structured in the following columns.

1. Macroprocess: biochemistry/immunochemistry or hematology;
2. Process: it reports the three main subprocesses (pre-analytical, analytical and post-analytical phases) identified within a laboratory;
3. Macrophase: for each process, the macro-activities required daily to run samples;
4. Mesophase: detailed individual activities.

**FIGURE 1:**
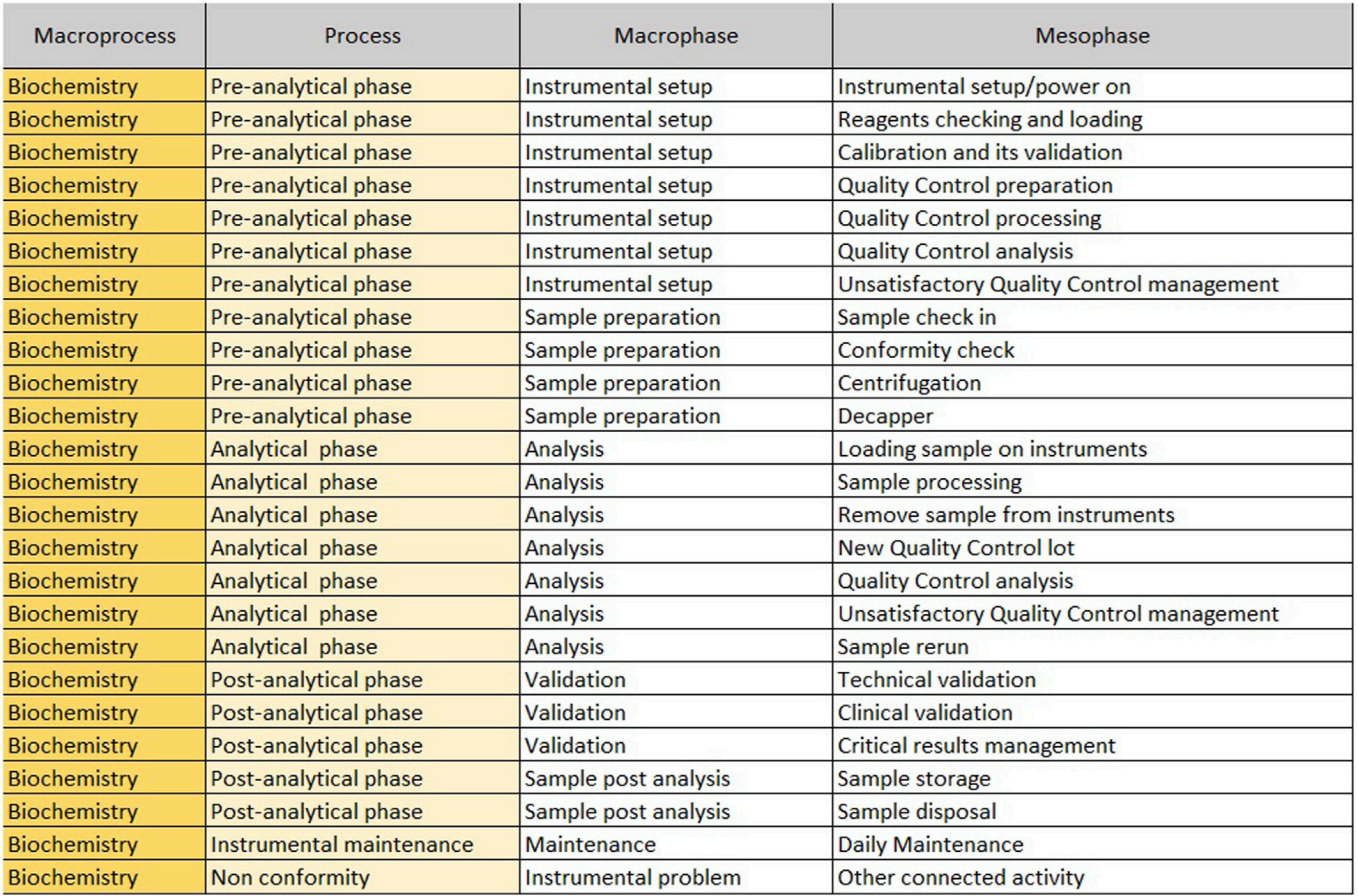
Mesophase time entry, biochemistry.

**FIGURE 2:**
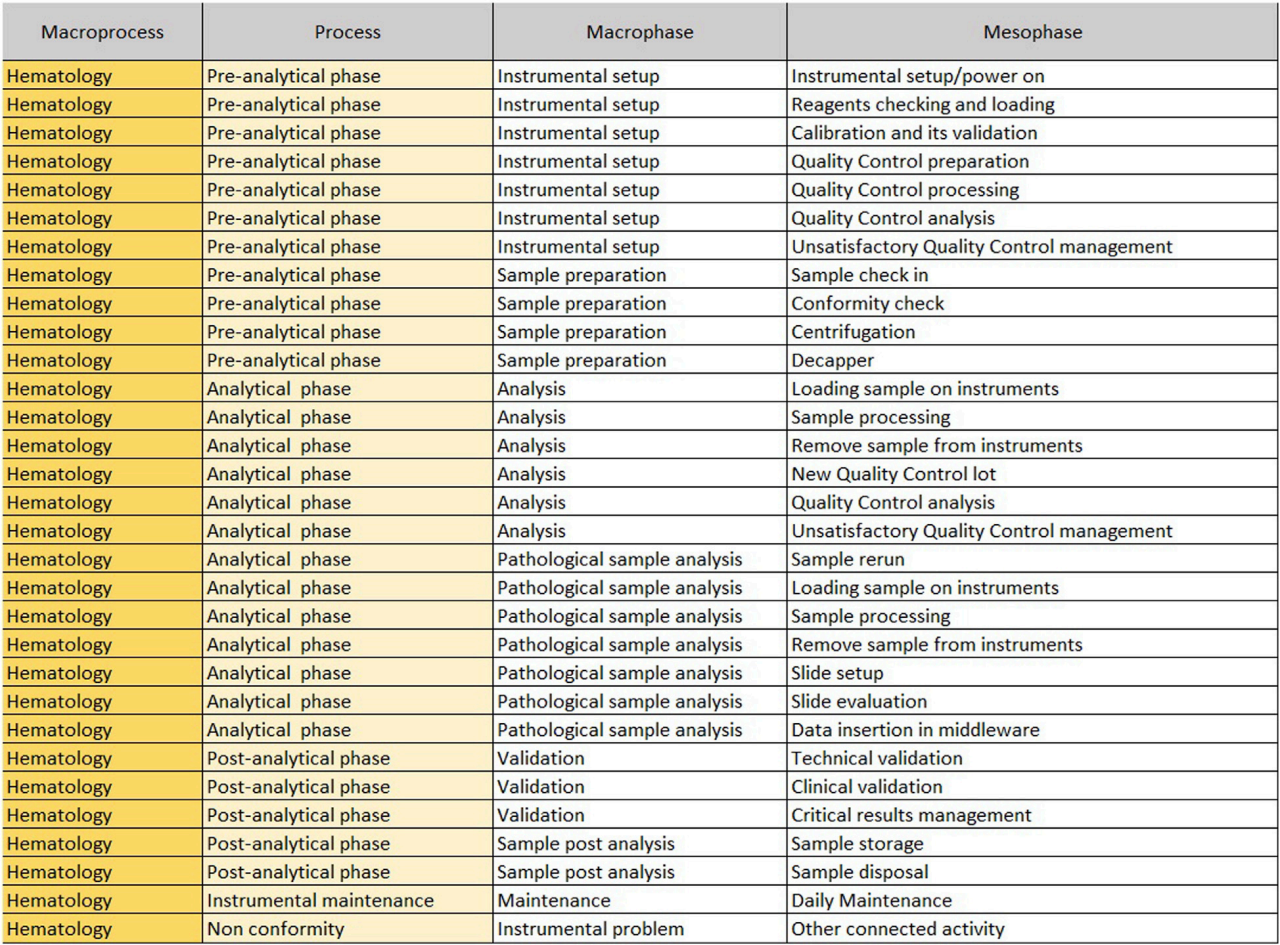
Mesophase time entry, hematology.

**FIGURE 3:**
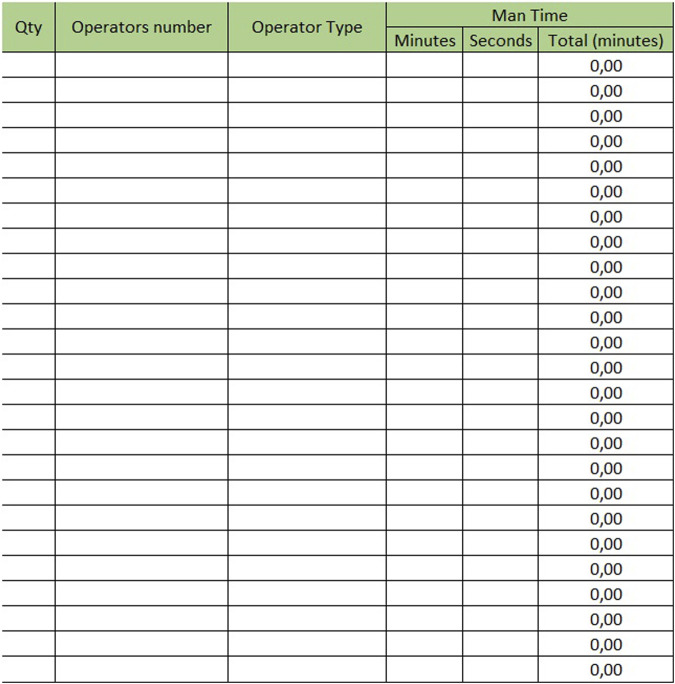
Mesophase time entry.

The sheet with “Mesophase Time Entry” for biochemistry/immunochemistry is shown in Figure 1, while the one for hematology is shown in Figure 2. Instead, Figure 3 shows the “Mesophase Time Entry” columns to be filled in, which are common for both sectors.

1. Qty: the number of units involved during the timing measurement of the single mesophase;
2. Operator number: the number of operators involved in the individual mesophase during the timing survey;
3. Operator Type: the professional figure performing the mesophase (Biomedical Scientist, Health Worker, Biologist or Doctors);
4. Man Time: the time, measured in minutes and seconds, to carry out the individual mesophase.

**FIGURE 4:**
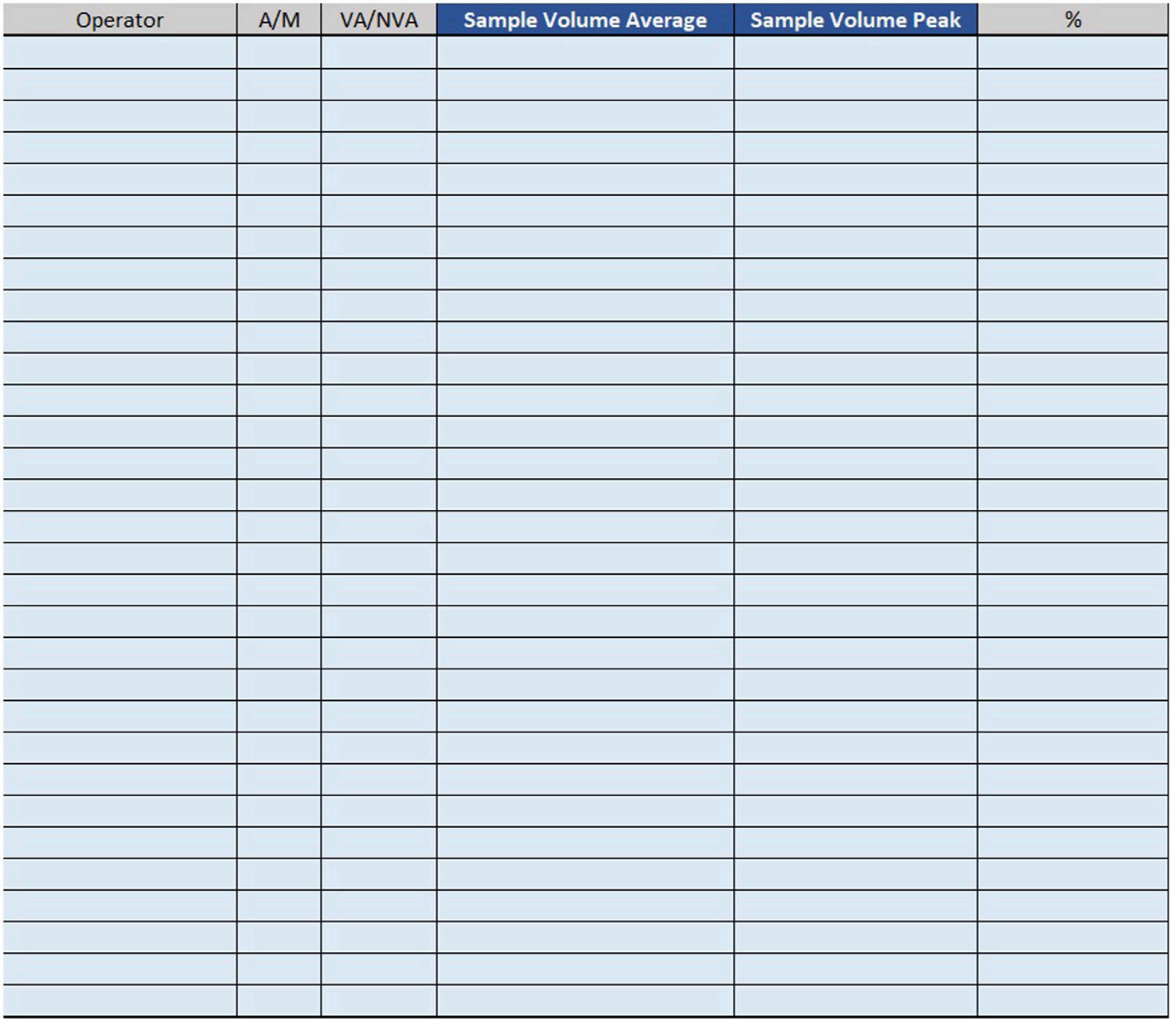
Official coding.

For example, if the time taken by two Biomedical Scientist to uncork 50 samples is measured in the mesophase ‘uncorking’ during time recording, the value 50 must be entered in “Qty”column, the value two in the ‘Number of operators’ column and the measured time expressed in minutes in ‘Man time’ one. The format will automatically calculate the seconds it takes a single operator to uncork a single sample by dividing the time by 50 and then by 2. The parameters, obtained by mathematical formula, contribute to the compilation of the “Official Coding” sheet, where the final calculations are shown. In order to make the measurements as reliable as possible, it was agreed to take five or more measurements for each mesophase. It would be appropriate to take measurements on different days, at different times of the day, and with different operators. This would allow more variables to be included in the measurements: from those related to analytical problems, to those due to the different skills of the personnel involved. In the “Official Coding” sheet, the first four columns show the same activities as in the previous one shown in Figures 1, 2. There are then other columns, shown in Figure 4, in which there is additional information useful for the final calculation.

− Operator: type of operator performing the mesophase;
− *A/M*: manual or automated activity. This parameter is not strictly part of the calculation of required Full Time Equivalent (FTE), but it is useful for benchmarking, when a comparison should be made between different laboratories to see which activities could push toward more automation.
− *VA/NVA*: Value-added activities (VA) are those that allow the product to be transformed, for example, slide evaluation or quality control verification, where the human figure is an added value. Non-value-added activities (NVA), are not value-added to the human figure, even though they are critical to the process.
− Sample Volume Average: average daily number of units, based on at least monthly extraction.
− Sample Volume Peak: daily peak of units, for example, in a monthly extraction, the day of the month when there were the most units involved in the mesophase.
− %: percentage of samples involved in the measured mesophase.

In laboratories with a 7-day-a-week service, a monthly extraction of the daily average of samples would be misleading: Saturdays and Sundays, being considerably fewer samples than midweek days, would considerably lower the daily average. For these laboratories, it is recommended to complete two different “official coding” sheets: one for midweek days and one for weekends.

**FIGURE 5:**
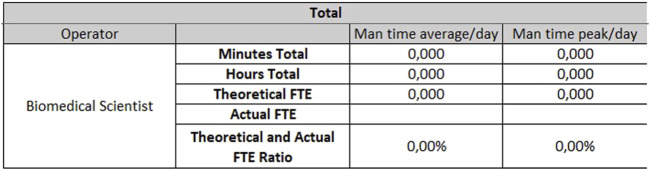
Official coding - Results chart.

Filling in all the data requested, The “Official Coding” sheets, after an automatic calculation, shows the results in a chart (Figure 5). It always refers to FTE: the number of full-time resources needed to perform the laboratory tests. To obtain the final value in FTE, the minutes per day devoted to that activity (obtained from the average of the five measurements and multiplied by the number of units) are summed for each individual mesophase. The value is not inclusive of the total number of hours needed to secure shift off, vacation, days of sickness and permit, but simply expresses the actual staffing requirements to carry out the activities. Therefore, it is necessary to calculate the total staffing.

**FIGURE 6:**
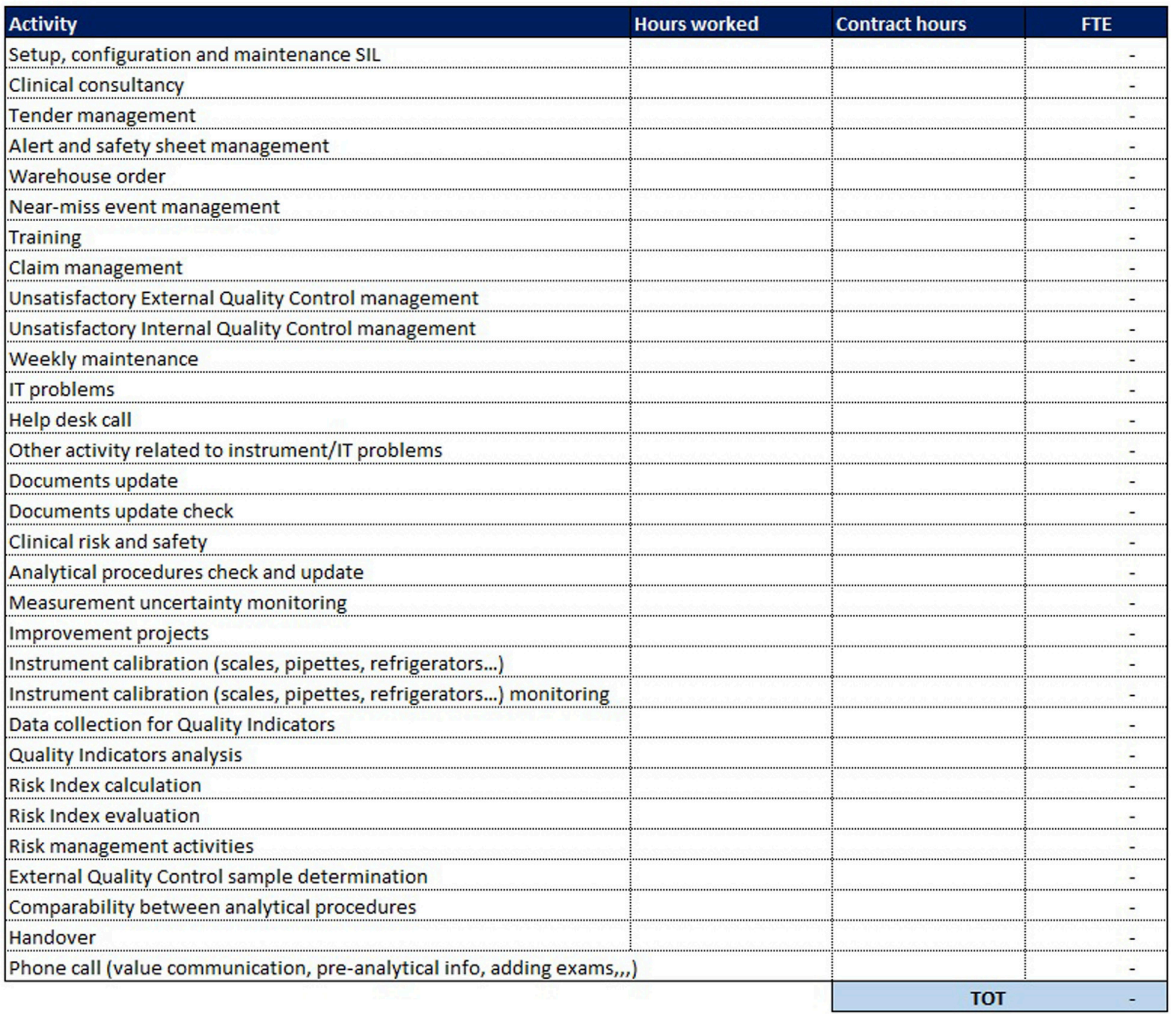
Extra production activities.

In addition to these two sheets, which are more purely dedicated to analytical activities, a third sheet is added for “Extra production activities”: here are all those so-called “indirect” activities, complementary to the performed service but not directly related to the analysis of the sample. These activities are essential to ensure the quality of the process and the reliability of laboratory performance. It is necessary to indicate, for each activity, the monthly number of hours devoted to that activity and the number of monthly hours provided by the national collective contract. The sheet dedicated to extra-production activities is the same for both analytical lines (biochemistry/immunochemistry and hematology) and is shown in Figure 6.

In the last column, the ratio is made between the monthly hours devoted to that activity and the hours stipulated in the contract; from their sum is derived the number of FTEs needed to carry out extra-production activities.

## 3 Results

All required data were collected and put into the proposed format. The application of this methodology makes it possible to calculate the need for Biomedical Scientist for the two areas, expressed in FTE.

**FIGURE 7:**
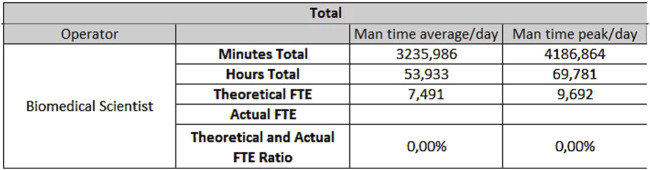
Results for biochemistry - Monday through Friday.

**FIGURE 8:**
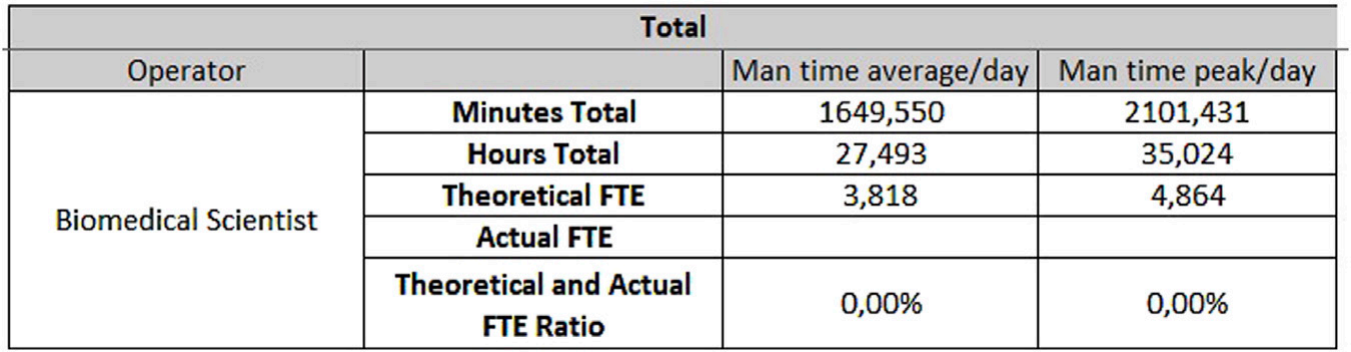
Results for biochemistry - Saturday and Sunday.

For the analytical line of clinical biochemistry and immunometry, 0.87 FTEs are required for extra-production activities, 7.49 FTEs for activities taking place on Monday through Friday (Figure 7) and 3.81 FTEs for Saturday and Sunday (Figure 8).

**FIGURE 9:**
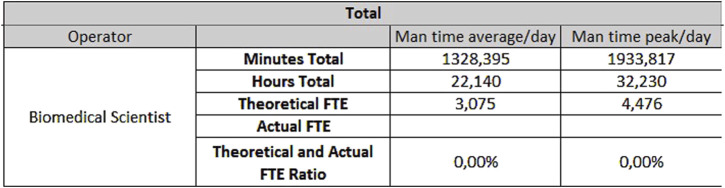
Results for hematology - Monday through Friday.

**FIGURE 10:**
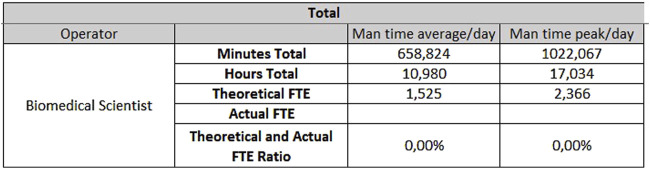
Results for hematology - Saturday and Sunday.

The hematology sector of the same laboratory needs 0.33 FTEs for extra-production activities, 3.07 FTEs for activities carried out from Monday to Friday (Figure 9), and 1.53 FTEs for Saturdays and Sundays (Figure 10).

The data shown are obtained on the basis of the daily average of samples and units, but if it is considered the daily peak, the FTEs needed would be:

- For hematology, 4.48 FTEs from Monday to Friday, and 2.37 FTEs for Saturday and Sunday;
- For biochemistry, 9.69 FTEs from Monday to Friday and 4.86 FTEs for Saturday and Sunday.

**FIGURE 11:**
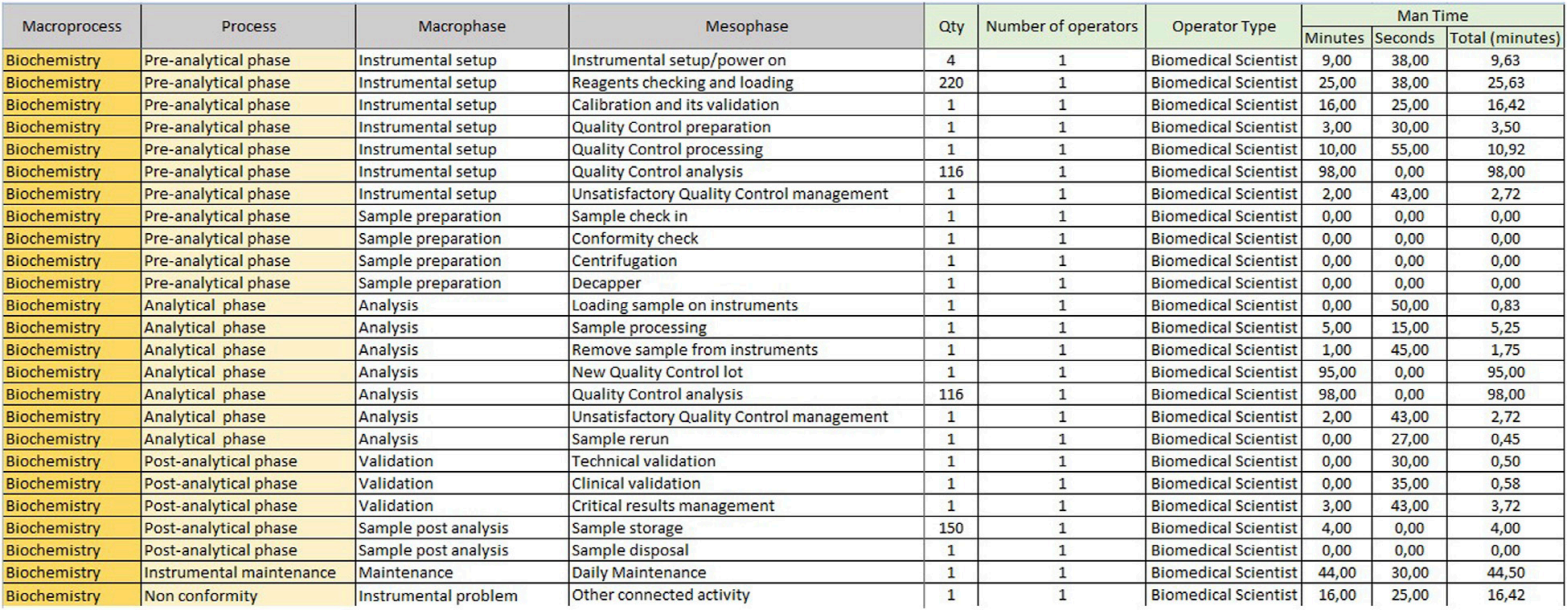
Example of a completed table of “Mesophase Time Entry” for the biochemistry sector.

**FIGURE 12:**
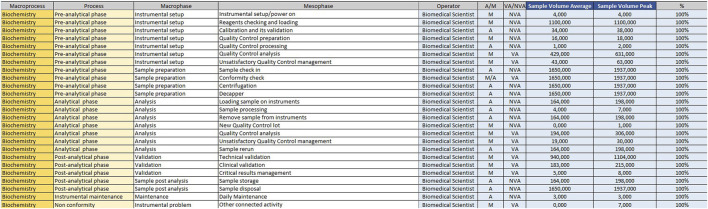
Example of a completed table of “Official Coding” for the biochemistry sector.

A prototype of the tables already completed for the biochemistry sector, to concretely demonstrate how the initial data are transformed into the final FTE count, is shown in Figures 11, 12.

## 4 Discussion

Applying this methodology allowed the calculation of biomedical staffing requirements for the two areas, expressed in FTEs. Until now, there was no model that could be used to estimate the need for Biomedical Scientist in such an analytical way: this is undoubtedly a first achievement of the project. Only applying this model to the other analytical lines that make up the Corelab area it will be possible to estimate the FTEs need for the entire area and draw the appropriate staffing considerations, as with the data to date, it is not possible to assess the congruence of the assigned staff. The sheet automatically calculates the ratio between theoretical and actual FTE.

The methodology made it possible to obtain an accurate value, but the project was not limited to the mere use of the format: a complex analysis was carried out in order to highlight the strengths and critical issues, which need to be solved for the methodology to be reliable.

The first advantage emerged is that a data, expressed in FTEs, is obtained and it represent an objective quantification of the need, being generated from a timely numerical survey. Using this value, it can be understood whether the level of staffing is adequate, whether it is oversized or undersized. In the latter case, it is possible, thanks to this format, to see exactly how much staffing is lacking and especially which area needs to be strengthened. Another strength of the methodology is the careful evaluation and enhancement of extra-production activities. These, as an integral part of the process, demonstrate how the role of the Biomedical Scientist and the activities performed, have changed over time: from a simple “operator”, who performs laboratory tests, the Biomedical Scientist has become a “professional” manager and guarantor of the quality of the process. The “Extra-Production Activities” sheet was designed to be extremely flexible: there is, in fact, the possibility of adding endless extra-production activities, so that each laboratory can somehow “personalize” it since each reality is deeply different in many aspects. In addition, the filling format showed how the demand for services is extremely variable. The format also requires among the data, a daily peak activity, allowing to predict how the organization should respond to fluctuations in the demand for laboratory tests. It is therefore necessary to adopt strategies in order to allocate resources without sacrificing the quality of the data and its timely reporting, especially in laboratories that work in emergency regime. One of the added values of this methodology is that the “Official Coding” sheet reports which activities are value-adding and which are non-value-adding. Those activities that add value to the product can be identified and improved; those that do not can be reassessed for better efficiency. This is possible because the methodology, based on ABM, places the focus on activity management in order to improve patient value. One of the advantages is that the format allows reasoning, not only on staffing, but also on activities and processes for their continuous improvement. This method can aid to prioritize the recruitment of Biomedical Scientist in certain laboratory sector, to optimize utilization of the existing workforce and to reallocate them based on the existing workload. The results might inform economic decisions as the ABM could facilitate the precise identification of inefficiencies, including the costs associated with non-value-added activities. The proposed format can also be used to calculate management staffing requirements, whether medical or biologist.

Comparing with the WISN method to assess the health workforce requirements, the ABM proposed method is easier to apply, more straightforward and structured. The “Workload indicators of staffing need. User’s manual” (World Health Organization, 2010) is a generic, non-laboratory-specific manual of 74 pages, so in addition to the already lengthy data collection work, it is also required to adapt the WISN method for laboratory context and to define all the current workload components of laboratory cadre. On the other hand, in the proposed format, which is specific not only for the laboratory, but also for a precise Sector of It (biochemical o hematology), the activities are already classified and summarized in two simple tables; it is only necessary to collect the timings and enter them, and the FTE calculation is automatically generated.

In contrast, several critical issues emerged in the compilation of the format. A first issue, common to all ABM applications, concerns data collection. In fact, it is essential to be punctual in the collection of data because the result can vary greatly, leading to wrong considerations. The problem does not only concern who to commission the survey, but also the huge amount of time it takes to obtain the data. The creation of the model and the identification of supporting tools must ensure that the effort for its compilation does not outweigh the achievable benefits. Considering that continuous technological innovations have affected almost all laboratory sectors in recent years, and that these proceed hand in hand with organizational ones, laboratory managers may frequently be required to review workloads and to re-collect timelines and data to update the format. Once understood the underlying methodology and how the format works, the model can be dynamically updated following significant technological or organizational changes. Moreover, the fact remains that not all activities can be quantified with a point-in-time measurement, but timeframes can simply be estimated, and if the estimate does not exactly match reality, the result may change even considerably. In addition, not for all laboratories the activities are divided equally and often, especially where there is a very high level of automation as in the Corelab area, there is an extremely relevant integration of processes. It must be kept in mind that more detail provides more information, making measurement more accurate and precise, but it makes surveying more complex and time-consuming. There is consequently the need to find the correct balance between an analytical format, allowing for truthful surveying, and a user-friendly one that does not take up too much time in its application, otherwise it may be unsuccessful. The format, on the surface quite simple, straightforward, and intuitive, turned out in practice to be difficult to fill out and not very clear, as certain required fields may be subject to different interpretations by those called upon to fill it out. It certainly needs to be accompanied by a detailed explanation so that there are no gross errors in filling in due to a lack of understanding of what is required. In conclusion, it would be desirable to improve the format by aiming for a uniform, reliable and reproducible interpretation in any center. Accordingly, the format represents a project that is still in its embryonic stage: while its compilation has revealed some of its limitations, it bodes well regarding future prospects if these critical issues are resolved. Regarding the format development, it is necessary to work towards greater simplification, applicability and reliability. However, there are broad prospects and plenty of room for improvement: formats dedicated to the other areas of clinical pathology laboratory (coagulation, toxicology and pharmacology, proteins, and allergology) need to be improved. Further broadening the perspective, it would be desirable to extend the methodology to other diagnostic disciplines of Laboratory Medicine such as microbiology, pathological anatomy and genetics, disciplines that nowadays have the same problems in the calculation of personnel requirements.

In conclusion, the use of Activity Based Management as a methodology for analyzing and evaluating the staffing needs, is suitable for the project objective: it perfectly marries the purpose of combining effectiveness and efficiency. It aspires, in fact, to achieve continuous improvement in efficiency by aiming to optimize processes and quantifying the personnel to be dedicated to them. It also allows management effectiveness by ensuring adequate workloads that are essential to guarantee high quality of laboratory data. The added value of the project is that it can be considered a valid tool for laboratory managers to calculate biomedical staffing needs but also had a relevant impact on the analysis of the processes, their efficiency, and their possible improvement. Activity Based Management focuses on the main objective of any clinical laboratory: to create value for the patient by supporting diagnostic and treatment pathways through safe and reliable laboratory data, that cannot disregard the correct allocation of human resources.

## Data Availability

The raw data supporting the conclusions of this article will be made available by the authors, without undue reservation.
